# Comparison of AceScope With Tube-Channelled Blade Versus McGRATH MAC Video Laryngoscope With Non-channelled Blade in a Difficult-Airway Manikin Model

**DOI:** 10.7759/cureus.100407

**Published:** 2025-12-30

**Authors:** Tomomi Ishii, Takashi Kondo, Sachiko Otsuki, Yuki Ota, Soshi Narasaki, Yukari Toyota, Noboru Saeki, Yasuo M Tsutsumi

**Affiliations:** 1 Department of Anesthesiology and Critical Care, Hiroshima University Hospital, Hiroshima, JPN

**Keywords:** channelled blade, difficult-airway management, manikin study, tracheal intubation, video laryngoscope

## Abstract

Background and Objectives: The AceScope (Ace Medical Co., Ltd, Seoul, Republic of Korea) is a new Macintosh video laryngoscope equipped with a tube-channelled type blade. This study aimed to evaluate the effectiveness of the AceScope with a tube-channelled type blade compared to the McGRATH MAC video laryngoscope (Medtronic plc, Galway, Ireland) with a non-channelled type blade in a difficult airway model.

Materials and methods: Twelve experienced anesthesiologists performed tracheal intubation on a difficult airway manikin using both devices. The primary outcome measured was intubation time. Secondary outcomes included user satisfaction, intubation success rate, and dental damage rate.

Results: The AceScope with a tube-channelled type blade significantly reduced intubation time compared to the McGRATH with a non-channelled type blade (16 vs. 19 seconds, p=0.04). There were no significant differences in user satisfaction or intubation success rates between the two devices, and no tooth injuries were observed with either device.

Conclusions: The AceScope with a tube-channelled type blade is more effective in reducing intubation time in difficult airway scenarios than the McGRATH with a non-channelled type blade. However, these findings are limited to manikin models.

## Introduction

Both in the operating room and outside of it, the video laryngoscope has been shown to increase the success rate of tracheal intubation, compared to the direct laryngoscope [1,2]. Since Macintosh-type video laryngoscopes can be used in the same manner as the direct laryngoscope, several recommendations suggest that video laryngoscopes should be used as the first choice for tracheal intubation, instead of direct laryngoscopes [3-5].

For the management of a difficult airway, a video laryngoscope is even more important [6] because it has a higher initial intubation success rate and better vocal fold visibility than a direct laryngoscope [7]. Therefore, a video laryngoscope is an essential device for safe tracheal intubation in a patient with a difficult airway.

Video laryngoscopes are classified by blade into three types: (i) the Macintosh-type, (ii) the extra-curved blade type, and (iiii) the tube-channelled type [6]. When using the Macintosh-type and the extra-curved blade, it is necessary to pre-shape the tracheal tube with a bougie or stylet to be guided from the mouth to the glottis [6]. In contrast to the non-channelled type blade, a tracheal tube is pre-attached to the blade when using the tube-channelled type. Thus, manipulation of the tube is minimized in the upper airway. As a result, the time required for intubation by the tube-channelled type blade is expected to be reduced, as compared to other types of blades. However, the effectiveness of the tube-channelled type blade has not been fully established [8].

**Figure 1:**
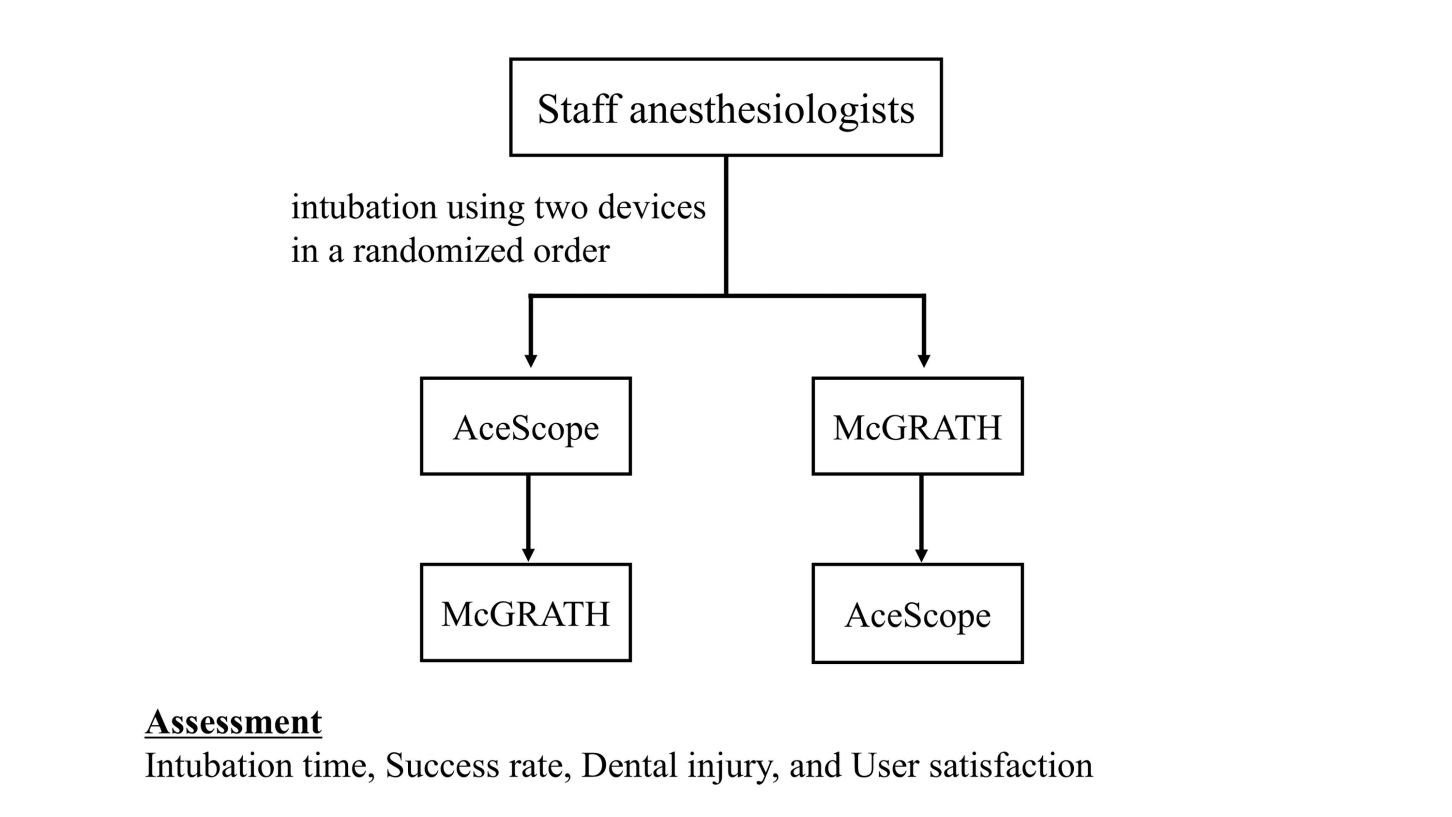
Schematic overview of the study design Each anesthesiologist performed tracheal intubation on a difficult-airway manikin using two devices in a randomized order: a Macintosh-type videolaryngoscope with a tube-channelled blade and a conventional Macintosh-type videolaryngoscope with a non-channelled blade. Intubation time, success rate, tooth injury, and user satisfaction were assessed for each device.

The AceScope (Ace Medical Co., Ltd, Seoul, Republic of Korea), a new Macintosh-type video laryngoscope, comes with a guiding channel blade. The guiding channel blade is a type of tube-channelled blade, used by pre-attaching a tracheal tube to a Macintosh-type blade (Figure 1). The AceScope is the first Macintosh videolaryngoscope equipped with a tube channel blade. Its usefulness should be evaluated in comparison to conventional devices.

In a patient with a difficult airway, the intubation time should be shorter because of the higher risk of hypoxia [9]. It was hypothesized that the feature of the guiding channel blade may shorten the intubation time, compared to the common Macintosh-type blade, in the setting of a difficult airway. This study, therefore, aimed to evaluate whether the AceScope with a guiding channel blade reduces intubation time and improves procedural performance in a difficult-airway manikin model.

## Materials and methods

Study design and site of study

This was a single-center, randomized, crossover manikin study conducted at Hiroshima University, Hiroshima, Japan. The study was approved by the Epidemiological Research Ethics Review Committee of Hiroshima University (Permit No. E2023-0127, October 4, 2023). 

Staff anesthesiologists performed tracheal intubation on a difficult-airway model manikin using two different devices: the AceScope and the McGRATH™ MAC video laryngoscope (Medtronic plc, Galway, Ireland). The participants were split into two groups: one group performed on the AceScope first and then moved on to the McGRATH (Medtronic plc, Galway, Ireland) (Group A), and the other performed on the McGRATH first and then moved on to the AceScope (Group M). Intubation time, success rate, tooth injury, and user satisfaction were assessed for each device (Figure 1).

Group allocation of participants

All participants had more than five years of experience in anesthetic practice, had performed over 100 tracheal intubations, and had used a video laryngoscope, including the McGRATH, on more than 50 occasions.

A pilot study was conducted before the main investigation with 10 participants. It was found that the average intubation times for both devices on 10 participants were 16±2.1 seconds for the AceScope and 26±5.4 seconds for the McGRATH (Table 1). These preliminary findings supported the feasibility of comparing the two devices in the main study.

**Table 1:** Preliminary results on intubation time obtained from a pilot study conducted with 10 participants

Parameter	AceScope	McGRATH
Intubation time (seconds), mean±SD	16±2.1	26±5.4

Following the results of the pilot study, JMP Pro version 17 (JMP Statistical Discovery LLC, Cary, North Carolina, United States) was used to calculate the required sample size. Sample size calculations resulted in nine participants per group. A type 1 error of α=0.05, and a power of 0.90 were used. Considering a possible 25% dropout rate, 12 participants were enrolled. All endpoints of this study were assessed for each device.

Intubation and manikin setup

**Figure 2:**
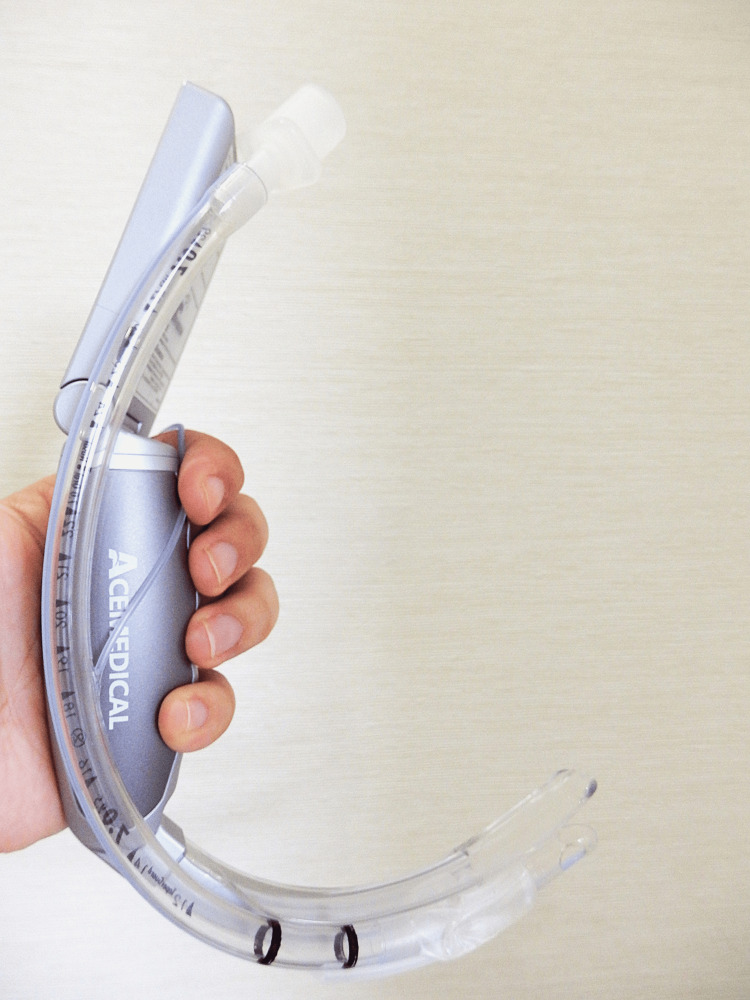
The AceScope (Ace Medical Co., Ltd, Seoul, Republic of Korea) with a guiding channel blade Image Credit: Tomomi Ishii

Tracheal intubation was performed using two different devices in random order as per the allocated group. Before this study, the participants were trained with both devices for five minutes on the difficult-airway model used for the present study. The AceScope (Figure 2) was used with a tube-channelled type blade. The McGRATH was used with a non-channelled Macintosh-type blade.

Blade size #3 and an internal diameter of 7 mm tracheal tube were used for both devices. For intubation with the McGRATH, a plastic (non-metallic), malleable stylet was used. The degree and shape of pre-curving of the tracheal tube were left to the discretion of the intubator.

A single individual was responsible for all intubation time measurements. The same person was the assistant for intubation. The intubator was informed beforehand that the assistant would provide support according to the intubator's instructions, following the same predefined sequence of assistance for all intubations, to minimize any effect on intubation time.

The details of the assistance included handing the device and intubation tube to the intubator, removing the stylet (Group M only), inflating the cuff with air, and handing the bag-valve mask to the intubator. The assistant passed the device and tracheal tube, removed the stylet, and performed other tasks according to the intubator's instructions.

**Figure 3:**
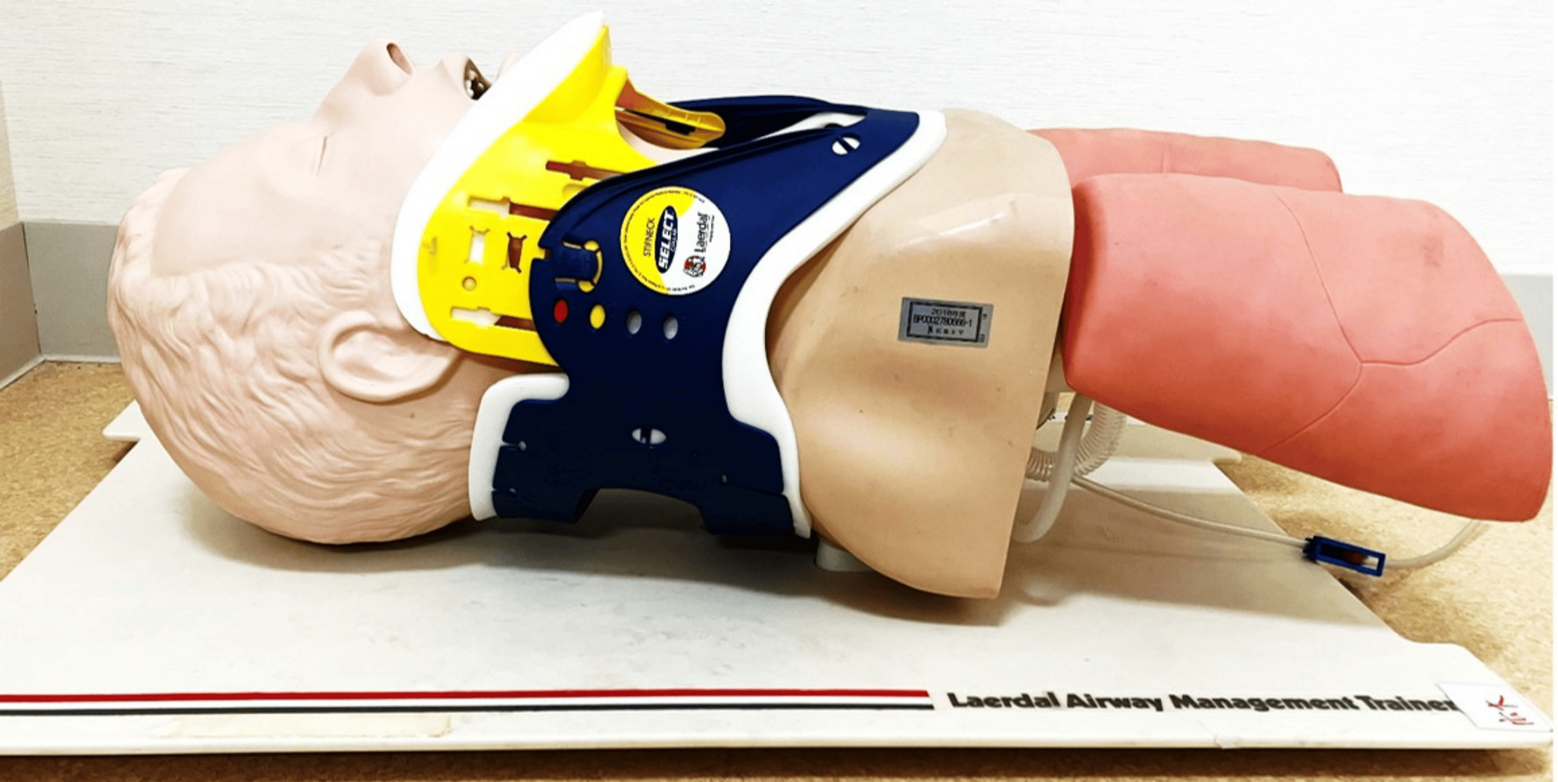
The Laerdal Airway Management Trainer with the Stifneck Select Collar Image Credit: Tomomi Ishii.

The difficult-airway model was set up with a cervical collar on the Laerdal Airway Management Trainer (Laerdal Medical, Stavanger, Norway). The Stifneck Select collar was used and fixed in the "regular" position. The manikin was placed on a non-adjustable desk with a height of 70 cm. This model was restricted to head retroflection and mouth opening (Figure 3).

Endpoints and data analysis

Intubation time was defined as the duration from the opening of the mouth to ventilation. Ventilation was confirmed by connecting a bag-valve mask to the intubation tube. The manikin was considered ventilated when lung inflation was directly observed. Failed intubation was defined as an intubation time longer than 120 seconds or esophageal intubation.

The primary endpoint of this study was intubation time. Secondary endpoints included the satisfaction of the intubator, the success rate of intubation, and the rate of tooth injuries. Tooth injuries were represented by the clicking sounds of the teeth of the manikin. Satisfaction with usage was rated on a 5-point scale. A score of 1 (worst) indicated "never use again," 3 (normal) indicated "same level as usual," and 5 (best) indicated "very satisfied." Immediately after the study was conducted, the questionnaires were distributed and completed anonymously in an unsupervised setting. The above endpoints were compared between Group A and Group M.

Continuous variables were compared using the Mann-Whitney U test, and Fisher’s exact test was used to compare categorical variables. The findings were considered statistically significant when P< .05. All statistical analyses were performed using Graphpad Prism 9.0 (Dotmatics, Boston, Massachusetts, United States).

## Results

A total of 12 anesthesiologists participated in this study. The crossover design ensured that six participants performed intubation using the McGRATH after the AceScope, while the other six performed intubation using the AceScope after the McGRATH, thus minimizing the influence of learning effects and inter-operator variability.

As shown in Table 2, the intubation time differed significantly between the two groups. The AceScope-first group (Group A) demonstrated a shorter intubation time compared to the McGRATH-first group (Group M), indicating that device sequence influenced procedural speed under the study conditions. This difference in intubation time represents the primary outcome of the present study. In contrast, no significant differences were observed between the groups with respect to subjective procedural assessment. Satisfaction scores evaluated on a five-point scale were comparable in groups A and M, indicating that both devices provided a similar level of operator satisfaction during intubation.

**Table 2:** Comparison of the satisfaction for intubations, the success rate of intubations, and the rate of tooth injuries between the two groups (N=12) Intubation time and satisfaction scores were analyzed using the Mann–Whitney U test, with Z values shown. Success rate and tooth injuries were compared using Fisher’s exact test. A Macintosh-type videolaryngoscope equipped with a tube-channelled blade was used with the AceScope (Ace Medical Co., Ltd, Seoul, Republic of Korea), and a conventional Macintosh-type videolaryngoscope with a non-channelled blade was used with the McGRATH MAC video laryngoscope (Medtronic plc, Galway, Ireland). IQR: interquartile range

Parameters	AceScope	McGRATH	Test statistic	P-value
Intubation time (seconds), mean (IQR)	16 (13.3-21)	19 (18-21.8)	Z=1.98	.04
Satisfaction for intubation (1-5), mean (IQR)	3 (1-3.5)	3 (2-3)	Z=-0.52	.75
Successful intubation, n (%)	12 (100)	12 (100)		>.99
Tooth injuries, n (%)	0 (%)	0 (%)		>.99

Regarding procedural success and safety outcomes, successful tracheal intubation was achieved in all cases in both groups, demonstrating that both the AceScope and the McGRATH allowed reliable airway access in the study model. Furthermore, no tooth injuries or other observable complications were identified in either group.

## Discussion

In this study, the AceScope with a guiding channel blade reduced intubation time more effectively than the McGRATH with a non-channelled blade in the difficult-airway model. Both devices used in this study were Macintosh-type video laryngoscopes, so the technique for visualizing the vocal cords was similar. However, the AceScope with a guiding channel blade comes with a tracheal tube pre-attached to the blade. This feature allows continuous guidance of the tube to the glottis without the need for a stylet.

When using a non-channelled blade for difficult airways, it is recommended that a stylet be used to pre-shape the tube [6,10]. However, during the insertion of the tracheal tube from the oral cavity to the trachea, a "blind spot" exists where the tube is not visible in either direct or video view. Furthermore, strong forces generated during the removal of the stylet can result in soft tissue injuries [10]. These factors can complicate tube guidance. In contrast, with a tube-channelled blade, the tracheal tube is inserted simultaneously with the device. Consequently, there is no need to insert the tracheal tube in a "blind spot." The results of this study suggest that the AceScope with a channeled blade may be recommended in situations where avoiding soft tissue injury is crucial, such as in patients with difficult airways who are prone to bleeding. However, this study was unable to directly examine the presence or absence of soft tissue injury.

When AceScope with channelled blades is used in patients with difficult airways due to cervical fusion, the time from vocal cord visualization to tracheal tube insertion has been reported to be faster than when non-channelled blades are used [11]. This result means that a blind tracheal tube insertion in a difficult airway is not easy, suggesting that channelled blades may be useful. In addition, the difficult-airway model manikin used in this study not only restricted cervical flexion but also restricted the mouth opening, which reduced the maneuvering space in the mouth for the tracheal tube, making it even more difficult to guide the tube to the glottis with a non-channelled type of blade, even if the glottis was visible. For these reasons, the Macintosh video laryngoscope AceScope with a guiding channel blade (a type of tube-channelled blade) was associated with a lower intubation time than the McGRATH with a non-channelled blade.

This finding contrasts with previous reports that indicated that tube-channelled blades, when used with non-Macintosh video laryngoscopes such as King Vision® Video Laryngoscope (Ambu A/S, Copenhagen, Denmark), resulted in longer intubation times compared to non-channelled blades [12]. Additionally, when comparing King Vision with a tube-channelled blade and GlideScope with a non-channelled blade (Verathon Inc., Bothell, Washington, United States), it was evident that while the intubation times were similar, the non-channelled blade had a higher success rate of intubation [13]. When using a tube-channelled blade with a non-Macintosh video laryngoscope, it is not always possible to insert the tracheal tube into the trachea, even if the glottis is visible [8]. To optimize the field of view specific to the tube-channelled blade, it is necessary to train with techniques different from those used with a direct laryngoscope [6].

The Macintosh-type video laryngoscope has similar laryngeal deployment techniques to the direct laryngoscope. This similarity may make it easier to operate than previous non-Macintosh-type video laryngoscopes, and these factors may have influenced the outcomes of previous reports. In this study, there was no difference in user satisfaction between the two groups. In a previous study of non-Macintosh video laryngoscopes, it was reported that the tube-channelled blade did not necessarily make intubation easier for experienced intubators. Instead, experienced intubators felt that the tube-channelled blade made the intubation technique more difficult [12]. Since the AceScope is a Macintosh video laryngoscope, it can be used in the same way as a regular laryngoscope for intubation. Therefore, we consider that the use of the AceScope with a guiding channel blade does not cause any special difficulty for experienced intubators.

This study suggests that the AceScope with channelled blades may allow more rapid intubation in difficult airways. In the future, we aim to conduct similar studies in real patients with difficult airways and examine different airway conditions, such as those in children and severely obese patients.

This study has several limitations. First, this was a manikin study, and the findings may not fully reflect clinical performance in real patients. In addition, the difficult-airway model represented only one type of airway difficulty, characterized by restricted neck movement and limited mouth opening; therefore, the results may not be generalizable to other causes of difficult airway, such as obesity or anatomical airway variations. Second, all intubations were performed by anesthesiologists with extensive experience in tracheal intubation and use of the McGRATH, and thus the findings may not be applicable to less experienced intubators. Third, although the use of a single assistant for time measurement improved consistency, the assistant was not blinded to the device being used, which may have introduced measurement bias. Finally, while the study was adequately powered for the primary outcome of intubation time, the sample size was relatively small for detecting differences in secondary outcomes such as tooth injury and user satisfaction.

## Conclusions

In this study, the AceScope with a guiding channel blade demonstrated shorter intubation times compared to the McGRATH with a non-channelled blade in a difficult-airway model, while both devices maintained similar success rates and safety profiles. These findings suggest that the guiding channel feature may facilitate faster and more efficient airway management in challenging scenarios. Overall, the results highlight the potential benefit of channelled Macintosh-type video laryngoscopes in reducing procedural time without compromising safety, providing practical implications for clinicians managing difficult airways.
